# Why identification matters: an explorative study on six cases of family reunification

**DOI:** 10.1007/s00414-024-03163-w

**Published:** 2024-01-16

**Authors:** Lorenzo Franceschetti, Debora Mazzarelli, Chiara Ragni, Francesca Paltenghi, Andrea Pecoraro, Denise Albani, Roberto Giuffrida, Cecilia Siccardi, Nicolò Polizzi, Paola Di Simone, Annalisa D’Apuzzo, Daniele Mandrioli, Martina Buscemi, Marilisa D’Amico, Ilaria Viarengo, Cristina Cattaneo

**Affiliations:** 1 LABANOF, Laboratorio Di Antropologia E Odontologia Forense, Sezione Di Medicina Legale, Dipartimento Di Scienze Biomediche Per La Salute, Università Degli Studi Di Milano, Via Luigi Mangiagalli 37, 20133 Milan, Italy; 2 Dipartimento Di Studi Internazionali, Giuridici E Storico-Politici, Università Degli Studi Di Milano, Milan, Italy; 3 UNHCR, United Nation High Commissioner for Refugees, Rome, Italy; 4 Laboratorio Di Genetica Forense, Gabinetto Regionale Della Polizia Scientifica Di Milano, Milan, Italy; 5 Dipartimento Di Diritto Pubblico Italiano E Sovranazionale, Università Degli Studi Di Milano, Milan, Italy; 6 Laboratorio Di Genetica Forense, Gabinetto Regionale Della Polizia Scientifica Di Palermo, Palermo, Italy

**Keywords:** Humanitarian forensic sciences, Dead migrants, Family reunification, Identification, Human rights

## Abstract

The escalating phenomenon of migration, accompanied by a disturbing surge in associated tragedies, has persistently violated internationally protected human rights. Absence of physical evidence, namely the presence of adequately identified corpses, may impede the full enjoyment of human rights and—in some cases—the course of justice as it obstructs the initiation of legal proceedings against individuals implicated in causing such catastrophes. It also presents administrative obstacles, as death certificates are indispensable in legitimizing statuses like orphanhood and widowhood. Family reunification, particularly for orphans, plays a significant role for those attempting to reconnect with their relatives all over the world. Likewise, for mothers, the acknowledgment of their marital status or widowhood can be a pathway to regain their marginalized right to social life. To elucidate this issue, we analyzed six representative cases from the tragic October 3, 2013, shipwreck near the Italian island of Lampedusa, where 366 individuals were retrieved dead from the sea. These cases underscore the practical challenges involved, highlighting the compelling need for continued efforts to ensure that this burdensome problem transcends from being a mere ethical, moral, and legal discourse. Although considerable progresses, these cases also reveal that substantial work still lies ahead. There is a pressing need for improved mechanisms to certify kinship ties, which are often the limiting factor in many reunifications, and can hinder the granting of custody to children. The severity and far-reaching implications of this problem necessitate thoughtful attention and action, especially considering the ongoing escalation in migration and related fatalities.

## Introduction

Over the past years, there has been an exponential increase in the phenomenon of migration. Since the onset of the migration crisis, the Mediterranean Sea has been the site of a tragic and ongoing loss of life [1]. Even when these episodes are large in proportion, they are often ignored or treated inadequately, which constitutes a continuous trampling of internationally recognized and protected rights [2]. The need to identify the deceased migrants not only respects their dignity but also significantly impacts surviving family members’ lives, especially concerning legal matters like access to family reunification [3–5].

Given the unfortunate and frequent occurrence of migrant fatalities, the demand for comprehensive legal measures to ensure proper identification and subsequent family reunification has amplified. The complexity and difficulty of these cases are enhanced by the inherent issues of jurisdiction, the differing legal codes across countries, and the practical challenges of identifying deceased migrants [6, 7]. If there is no corpse, there can be no crime from a legal point of view. This means that family members lose the right to be civil parties in criminal proceedings against those allegedly responsible for the shipwrecks [1, 8, 9]. From an administrative point of view, in the absence of death certificates for parents or spouses, the administration of life involves serious delays and obstacles [1, 10]. Moreover, the lack of a death certificate for orphans of the migration phenomenon often means that they cannot reunite with relatives in Europe and may remain abandoned in countries of war. Meanwhile, their mothers, in the absence of marital or widow status, remain marginalized and deprived of their right to social life.

Starting from the shipwreck of October 3, 2013, near the Italian island of Lampedusa, where 366 bodies were recovered from the sea, we present six cases in which the recognition of orphan or widowhood status was essential to ensure the protection of the rights of children and widowed women. We discuss the critical practical issues, ensuring that this burdensome problem does not remain a mere ethical, moral, and legal consideration. These emblematic cases demonstrate that much has been done, but much more must still be done to address the severity of this worsening situation.

## Case presentations

Since 2007, the Laboratory of Forensic Anthropology and Odontology (Laboratorio di Antropologia e Odontologia Forense – LABANOF) of the University of Milan has been collaborating with the Office of the Commissioner of Missing Persons of the Italian Government (Ufficio del Commissario per le Persone Scomparse – UCPS). The aim of the collaboration is to guarantee the right to the identity of normal domestic unknown decedents found on national territory [11] and of migrants who died in shipwrecks in the Mediterranean Sea [12]. In particular, thanks to cooperation also with United Nations High Commissioner for Refugees (UNHCR), Italian Red Cross (IRC), International Organization for Migration (IOM) among the cases dealt with, it has been possible to obtain details of the motivations behind the request for death certificates. The cases presented below were selected as emblematic because of their characteristics that have allowed the legal issues to emerge which will be detailed below.

Genetic analyses, used to compare ante-mortem profiles or family DNA samples and post-mortem data, were carried out by the Regional Cabinet of the Scientific Police in Milan, which actively collaborates with LABANOF in the identification procedures. The protocols followed in order to achieve a positive identification are the same as those described in [13].

### Case 1

The first case involves a 13-year-old Eritrean boy who, following his parents’ separation, relocated with his mother to Sudan in a refugee camp. His mother attempted to reach Europe via a fishing boat, perishing in the process during the shipwreck of October 3, 2013. In 2016, the boy’s maternal relatives in Canada and Sweden managed to contact LABANOF staff. The ante-mortem material and the boy’s genetic sample were collected and compared with profiles extracted from the 366 recovered victims housed at LABANOF. The juxtaposition of the living mother’s photos with post-mortem material and subsequent genetic investigations proved crucial in establishing the familial link between the boy and the deceased woman. The resultant death certificate enabled the initiation of family reunification paperwork between the boy and his aunt in Sweden.

### Case 2

In 2013, an Eritrean child, residing with his mother in a Sudanese refugee camp, became fatherless following the maritime disaster off the coast of Lampedusa. The remaining family members attempted to seek assistance from the UNHCR, but they were unsuccessful as the man’s death certificate was required. In 2016, the IRC intervened and contacted the UCPS, soliciting aid for the family stranded in Sudan. Subsequent comparisons between the child’s genetic profile and those extracted from recovered shipwreck victims allowed the establishment of his status as an orphan and his mother’s as a widow.

### Case 3

In 2017, a migrant center in Uganda became the refuge settlement for a mother and her four children, aged 3, 6, 7, and 8. They were yet to hear from their father, who had departed in 2013 in pursuit of improved living conditions. The youngest child, born in 2014, was deprived of any direct familiarity with his father. The UCPS liaised with LABANOF to help resolve their stalemate. Blood samples from the children enabled the confirmation of their paternal lineage among the shipwreck victims of October 2013, facilitating the certification of their father’s demise and establishing their status as orphans and their mother’s as a widow.

### Case 4

An Eritrean mother and her two children lived as refugees in Sudan following the father’s departure and subsequent disappearance in the shipwreck of October 2013. The woman found herself neither being officially recognized as a widow nor having the legal freedom to work. In 2017, she reached out to the UCPS. Upon the arrival of the children’s toothbrushes and salivary swabs in Italy, a successful match with the man’s genetic profile was accomplished. This allowed the recognition of her status as a widow and the children as orphans.

### Case 5

Following their father’s disappearance in the 2013 shipwreck, three Eritrean children aged 11, 13, and 14, along with their mother, were refugees in Ethiopia. Despite some attempts to reach their maternal aunt in Australia, in 2021 their mother appealed to the 3 October Committee, an association of relatives of the shipwreck victims. The IOM became involved, allowing LABANOF to obtain the children’s biological samples. A match was confirmed between all three children and the genetic profile of one of the shipwreck victims, opening the pathway for a possible family reunification via a humanitarian visa.

### Case 6

The most recent case revolves around a 13-year-old boy left alone in Sudan following his father’s loss in the 2013 shipwreck. The mother intended to bring her son to Germany on refugee status, but she was unable to prove her husband’s death to the German government as required by law. The 3 October Committee acted as an intermediary, and LABANOF received a sample of the child for analysis in 2021. However, an identification was not possible, due to the low yield of postmortem DNA typing from the alleged father remains. The woman was requested to provide usable ante-mortem material of her husband for identification, but only low-quality photographs of the man were available. Currently, the case remains inconclusive, with an identity suspicion but no confirmation, rendering family reunification unattainable at this point, lacking the consent of the father as required document to allow his departure from Sudan.

## Discussion

The intersection of international migration law, international human rights law, and, as the European context, EU Law renders the legal aspects of family reunification for deceased migrants particularly intricate. Forensic science emerges as a crucial player in these circumstances, as it facilitates the legally necessary processes of identification [14–19]. This, in turn, allows the issuance of death certificates, which are indispensable legal documents, lacking sufficient flexibility in some national procedures.

The death certificate acts as proof of an individual’s demise, delineating the cause, location, and time of death [20]. This document’s importance is amplified in the context of migration, where conditions often complicate death confirmation and individual identification.

One of the critical implications of a death certificate is to determine the legal status of surviving family members. As evident in the presented cases, the certificate establishes the surviving spouse as a widow(er) and as unique exercising parental authority over the child/children and the children as orphans. Such a status change may afford them certain legal rights, protections, and benefits under both domestic and international law [21–23]. For instance, under Italian legislation, the loss of the right to inheritance (Art. 456, 462, 536, 547 of the Civil Code), may result in forcing children to work by forgoing schooling, thus being deprived of another fundamental right, that of education (Art. 34 of the Constitution; Law No. 296 of December 27, 2006) [1]. Meanwhile, their mothers, in the absence of marital or widow status remain marginalized and deprived of their right to social life [24].

In the context of family reunification, death certificates may reveal indispensable. Most jurisdictions require proof of death as part of the application process for family reunification. In the absence of a death certificate, surviving family members may find it challenging to prove their relationship with the deceased, impacting their reunification entitlement.^1^ When only one parent has died, as seen in some of the reference cases, a death certificate remains crucial. It establishes the surviving parent’s status as a single parent or widow(er), which can affect their rights and eligibility for benefits. Similarly, it affects the child’s status, which may impact their family reunification rights or other protections.

Going into the specifics, despite the common threads of the six cases, each case’s circumstances differ, affecting the process of acquiring a death certificate and initiating family reunification. From a technical perspective, in cases 1, 2, 3, and 5, ante-mortem material or biological samples (genetic and photographic comparisons) were used to identify the deceased. This led to the issuance of death certificates, enabling family reunification. This pattern indicates the importance of preserving and using any available material related to the missing person for identification procedures. Contrastingly, case 4 does not involve an immediate relative to provide biological samples. Instead, salivary swabs from the children and toothbrushes were used to establish the man’s genetic profile. Case 6 presents an ongoing situation where the identification of the deceased has not been confirmed. This case illustrates the challenges faced when ante-mortem material is limited or of low quality and emphasizes more the significance of innovative methods in death verification, especially when traditional means are inaccessible.

While all six cases demonstrate the importance of death certificates in family reunification, their differences highlight various obstacles encountered in acquiring these documents. These include the unavailability of high-quality ante-mortem material (case 6), the difficulty in establishing contact with authorities (case 2), and the challenge of providing proof of paternal connection in the absence of a formal relationship (case 3).

Each case is strongly affected by legal issues, influenced in particular by the country of asylum, the refugees’ status, the host country’s requirements, and the intervention of humanitarian organizations. For instance, the role of LABANOF, the IRC, and the UCPS was significant in establishing identity in most of the cases.

However, navigating international family reunification can be complex, given the disparate legal systems and immigration policies involved.

The first step in this process is to understand the laws and policies of both countries involved. This includes the immigration laws and family reunification policies of the host country (where the family member currently resides or wishes to migrate), as well as the emigration laws of the home country (where the family member currently resides or from where they will be migrating). The UNHCR^2^ and IOM provide resources and assistance to individuals navigating the family reunification process. Similarly, non-governmental organizations that specialize in immigration law can also be useful resources [21–23].

According to EU Directive 2003/86/EC of 22 September 2003,

Under European law, the right of family reunification is expressly recognized under the EU Directive 2003/86/EC, which states that minors are granted the right to family reunification provided that both parents, in the case of shared custody, give their consent (Art. 4 par. 4 lett. c and d). The proof of death is therefore fundamental to demonstrate the impossibility to acquire the consent of both parents. The presence of a death certificate is even more important in the case of the application of the special regime accorded by the Directive to unaccompanied refugee children; Art. 10 n. 3 (b) member States may in fact authorize the entry and residence for the purposes of family reunification of their legal guardian or any other member of the family, where the refugee has no relatives in the direct ascending line or such relatives cannot be traced.

In addition, according to a well-established case-law of the European Court of Human Rights (ECHR), the right to stay with the family in a country different from the one of origin is considered as an expression of the right to family life (protected by Art. 8 of the ECHR) and depends on several considerations to be balanced one against the other (see *Rodrigues da Silva and Hoogkamer v. the Netherlands*, no. 50435/99, § 39, ECHR 2006-I, and *Antwi and Others v. Norway*, no. 26940/10, §§ 88–89, 14 February 2012). However, when children are concerned, their best interests shall prevail on any other interest at stake. In fact, according to both Art. 3 of the 1989 UN Convention on the Rights of the Child and to Art. 24 of the EU Charter of Fundamental Rights, the right “to maintain on a regular basis a personal relationship and direct contact with both his or her parents” was furtherly postulated (see *Popov v. France*, nos. 39472/07 and 39474/07, § 139, 19 January 2012, *Berisha v. Switzerland*, no. 948/12, § 49, 30 July 2013). Also, as to the specific situation of refugees, like those concerning the present research, the ECHR affirmed that the family reunion is an essential element in enabling persons who have fled persecution to resume a normal life and that exists a consensus at International and European level on the need for refugees to benefit from a family reunification procedure that is more favourable than that foreseen for other foreigners (see *Tanda-Muzinga v. France*, no. 2260/10, 10 October 2014, §§ 73–75; and more recently *[GC], M.A. v. Denmark*, no. 6697/18, 9 July 2021, §§ 151–156).

Moreover, it is necessary to consider that the requirements related to immigration control must be balanced with the protection of the right to family unity. The Strasbourg Court has always affirmed (see *Cherif and others v. Italy*, 7 April 2009) that the ECHR does not guarantee the right to enter a particular country, but, when close family members live in the country where the foreigner intends to stay, the right to family life must be protected and balanced in a proportionate manner with the public security, pursuant to Art. 8(1) of the ECHR. The same principle has been affirmed by National Courts, such as that of Italy (It. Const. Ct. Sent. 203 of 2013).

The importance of safeguarding the unity of the family members especially when children are concerned is also stressed by several international legal instruments [25–27]. The Universal Declaration of Human Rights (Art. 16, Section 3) asserts “The family is the natural and fundamental group unit of society and is entitled to protection by society and the State.” The UN Convention on the Rights of the Child elaborates this right further in Art. 10, Subsection 1: “In accordance with the obligation of States Parties under Art. 9, paragraph 1, applications by a child or his or her parents to enter or leave a State Party for the purpose of family reunification shall be dealt with by States Parties in a positive, humane and expeditious manner.” Moreover, the process of identification aligns with the ethical mandate of respecting the deceased’s dignity and their right to identification, as stipulated in Principle 9 of the UN’s “Principles on the Effective Prevention and Investigation of Extra-legal, Arbitrary and Summary Executions.”

However, jurisdictional issues and differing national laws can present formidable challenges. Each country has unique immigration and asylum laws and policies concerning family reunification. These divergent legislations affect the surviving family members’ ability to join their relatives in another country. While each country’s laws and regulations regarding family reunification may vary, the universal significance of a death certificate as a critical legal document supporting family reunification is undeniable. The goal remains the preservation of the unity of the family, as a fundamental human right enshrined in International law. Therefore, it is crucial to prioritize efforts to identify deceased migrants and issue death certificates. This not only acknowledges and respects their humanity but also facilitates the necessary legal procedures for surviving family members, easing their path to family reunification and a more secure future.

## Conclusions

The identification of deceased migrants is not only a matter of human dignity and rights [28–31] but also plays a crucial role in the legal processes associated with family reunification. Death certificates play a vital role in asserting the familial status of survivors and facilitating their reunification processes. The cases explored in the present research illustrate the significant challenges that refugees and immigrants face when a loved one dies en route to their destination. The limitation behind many reunifications is the lack of opportunities to certify kinship ties, which can prevent children from being granted custody. The scale and serious consequences of this problem should be considered in view of the massive increase in migration and associated fatalities.

## Data Availability

Data availability possible on motivated request to the corresponding author.

## Footnotes

1 “Proving family links or dependency may be particularly difficult especially where documentary evidence such as passports, birth certificates, marriage certificates or other such documents are missing and/or hard to access. In the same way, lack of documentation in the case of informal adoption of non-biological children may also delay or lead to a rejection of reunification. In the case of single parent families, they may have to provide proof of the death of the other parent and/or of parental authority, which may not be possible where the other parent remains in a conflict zone or such administrative services are no longer available from the State or prohibitively costly,” UN High Commissioner for Refugees (UNHCR), Families together, December 2018, available at: https://www.unhcr.org/neu/wp-content/uploads/sites/15/2020/10/Familiestogether_20181203-FINAL.pdf [accessed 15 Oct 2023]; and para 7.7, UN High Commissioner for Refugees (UNHCR), The “Essential Right” to Family Unity of Refugees and Others in Need of International Protection in the Context of Family Reunification, January 2018, 2nd edition, available at: https://www.refworld.org/docid/5a902a9b4.html (accessed 15 Oct 2023).

2 https://help.unhcr.org/
